# Supplementary material for: Sub-chronic testosterone treatment increases the levels of epithelial sodium channel (ENaC)-α, β and γ in the kidney of orchidectomized adult male Sprague–Dawley rats
Source: PeerJ. 2016 Jun 30;4:e2145. doi: 10.7717/peerj.2145 (PMC4933084; doi:10.7717/peerj.2145)
Supplement: Data S2 — Western blot replicates for a, b and g ENaC. [file peerj-04-2145-s002.pptx]

## Slide 1
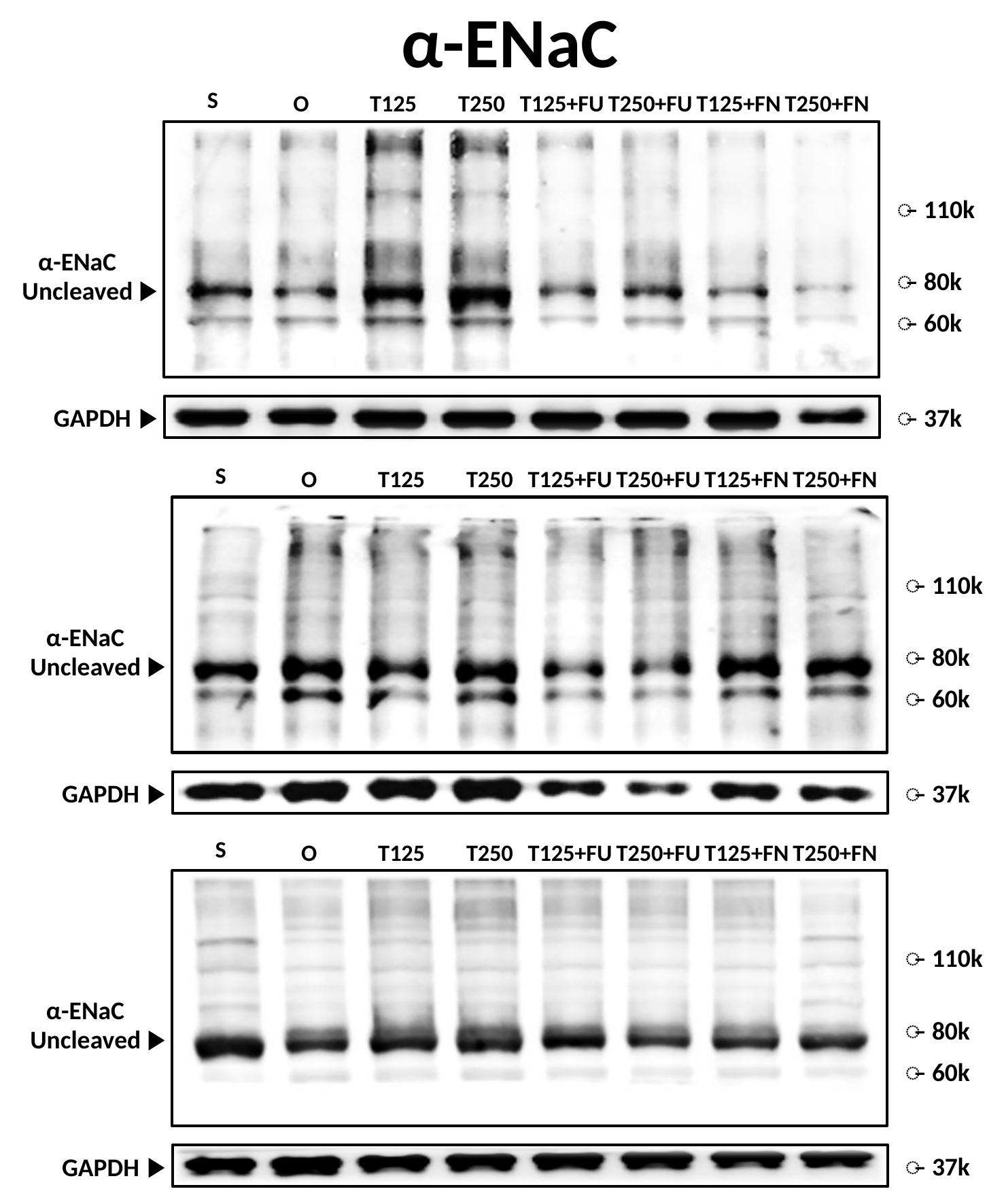

α-ENaC
S
O
T125
T250
T125+FU
T250+FU
T125+FN
T250+FN
̶ 110k
α-ENaC
̶ 80k
Uncleaved
̶ 60k
̶ 37k
GAPDH
S
O
T125
T250
T125+FU
T250+FU
T125+FN
T250+FN
̶ 110k
α-ENaC
̶ 80k
Uncleaved
̶ 60k
̶ 37k
GAPDH
S
O
T125
T250
T125+FU
T250+FU
T125+FN
T250+FN
̶ 110k
α-ENaC
̶ 80k
Uncleaved
̶ 60k
̶ 37k
GAPDH

## Slide 2
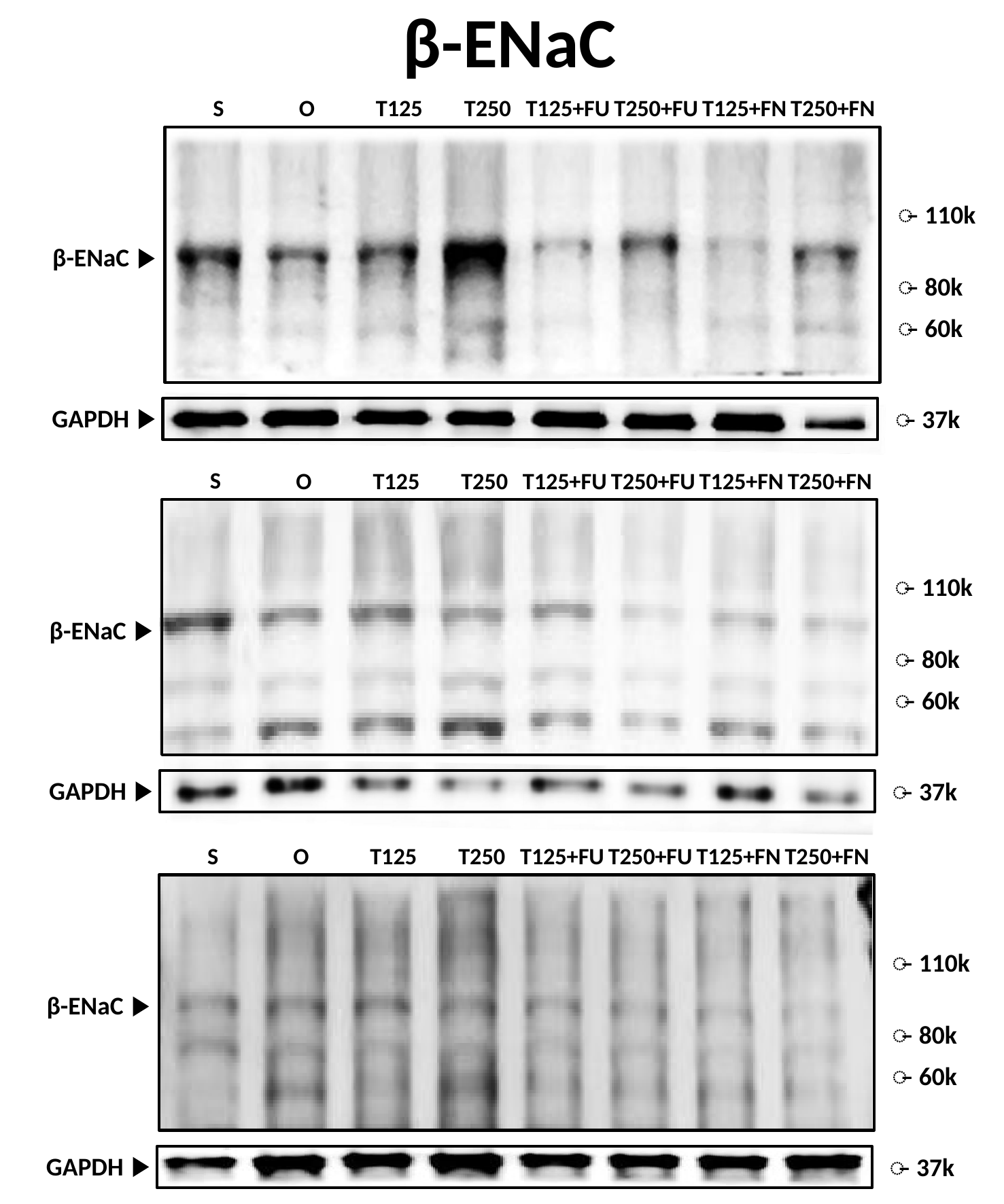

β-ENaC
S
O
T125
T250
T125+FU
T250+FU
T125+FN
T250+FN
̶ 110k
β-ENaC
̶ 80k
̶ 60k
GAPDH
̶ 37k
S
O
T125
T250
T125+FU
T250+FU
T125+FN
T250+FN
̶ 110k
β-ENaC
̶ 80k
̶ 60k
GAPDH
̶ 37k
S
O
T125
T250
T125+FU
T250+FU
T125+FN
T250+FN
̶ 110k
β-ENaC
̶ 80k
̶ 60k
GAPDH
̶ 37k

## Slide 3
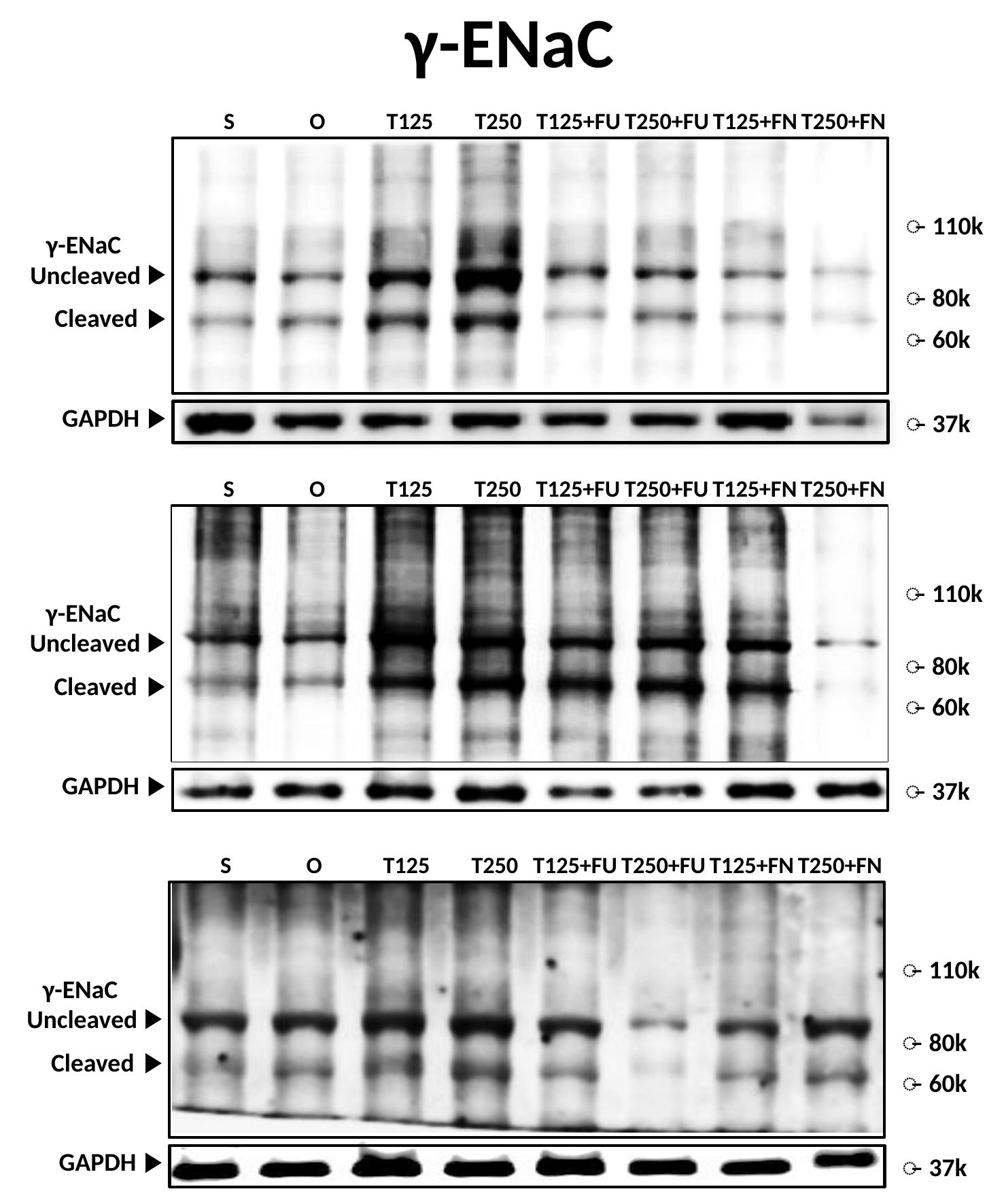

γ-ENaC
S
O
T125
T250
T125+FU
T250+FU
T125+FN
T250+FN
̶ 110k
γ-ENaC
Uncleaved
̶ 80k
Cleaved
̶ 60k
GAPDH
̶ 37k
S
O
T125
T250
T125+FU
T250+FU
T125+FN
T250+FN
̶ 110k
γ-ENaC
Uncleaved
̶ 80k
Cleaved
̶ 60k
GAPDH
̶ 37k
S
O
T125
T250
T125+FU
T250+FU
T125+FN
T250+FN
̶ 110k
γ-ENaC
Uncleaved
̶ 80k
Cleaved
̶ 60k
GAPDH
̶ 37k
